# Thrombocytopenia predicts mortality in Chinese hemodialysis patients- an analysis of the China DOPPS

**DOI:** 10.1186/s12882-021-02579-5

**Published:** 2022-01-03

**Authors:** Xinju Zhao, Qingyu Niu, Liangying Gan, Fan Fan Hou, Xinling Liang, Zhaohui Ni, Xiaonong Chen, Yuqing Chen, Keith McCullough, Bruce Robinson, Li Zuo

**Affiliations:** 1 Department of Nephrology, Peking University People’s Hospital, Unit 10C in Ward Building; 11 Xizhimennan Street, Xicheng District, Beijing, 100044 China; 2 Division of Nephrology, Nanfang Hospital, Southern Medical University, National Clinical Research Center of Kidney Disease, State Key Laboratory of Organ Failure Research, 1838 North Guangzhou Avenue, Guangzhou, China; 3 Division of Nephrology, Guangdong Provincial People’s Hospital, Guangdong Academy of Medical Sciences, Guangzhou, China; 4 Department of Nephrology, Renji Hospital, School of Medicine, Shanghai Jiaotong University, Shanghai, China; 5 Division of Nephrology, Ruijin Hospital, School of Medicine, Shanghai Jiaotong University, Shanghai, China; 6 Renal Division, Peking University First Hospital, Beijing, China; 7 Arbor Research Collaborative for Health, Ann Arbor, MI USA

**Keywords:** Thrombocytopenia, Mortality, Cardiovascular death, Hemodialysis, DOPPS

## Abstract

**Background:**

Hemodialysis (HD) patients have a higher mortality rate compared with general population. Our previous study revealed that platelet counts might be a potential risk factor. The role of platelets in HD patients has rarely been studied. The aim of this study is to examine if there is an association of thrombocytopenia (TP) with elevated risk of all-cause mortality and cardiovascular (CV) death in Chinese HD patients.

**Methods:**

Data from a prospective cohort study, China Dialysis Outcomes and Practice Patterns Study (DOPPS) 5, were analyzed. Demographic data, comorbidities, platelet counts and other lab data, and death records which extracted from the medical record were analyzed. TP was defined as the platelet count below the lower normal limit (< 100*10^9^/L). Associations between platelet counts and all-cause and CV mortality were evaluated using Cox regression models. Stepwise multivariate logistic regression was used to identify the independent associated factors, and subgroup analyses were also carried out.

**Results:**

Of 1369 patients, 11.2% (154) had TP at enrollment. The all-cause mortality rates were 26.0% vs. 13.3% (*p* < 0.001) in patients with and without TP. TP was associated with higher all-cause mortality after adjusted for covariates (HR:1.73,95%CI:1.11,2.71), but was not associated with CV death after fully adjusted (HR:1.71,95%CI:0.88,3.33). Multivariate logistic regression showed that urine output < 200 ml/day, cerebrovascular disease, hepatitis (B or C), and white blood cells were independent impact factors (*P* < 0.05). Subgroup analysis found that the effect of TP on all-cause mortality was more prominent in patients with diabetes or hypertension, who on dialysis thrice a week, with lower ALB (< 4 g/dl) or higher hemoglobin, and patients without congestive heart failure, cerebrovascular disease, or hepatitis (*P* < 0.05).

**Conclusion:**

In Chinese HD patients, TP is associated with higher risk of all-cause mortality, but not cardiovascular mortality. Platelet counts may be a useful prognostic marker for clinical outcomes among HD patients, though additional study is needed.

## Introduction

Mortality rate was much higher in Hemodialysis (HD) patients than that of individuals in the general population [1, 2]. According to the global burden of disease study in 2017, end stage kidney disease (ESKD) has become one of the three fastest growing causes of death in the world in the past 20 years [3]. Diverse risk factors for death have been identified including older age, arterial hypertension, diabetes mellitus, and ischemic heart disease and so on [4]. Our team’s research results suggested that platelet counts might be a potential risk factor (unpublished data). However, few studies have examined the association of platelet count with all-cause and cause-specific mortality in HD patients [5, 6].

Platelet count is a component of a routinely measured clinical assay. Platelet plays a crucial role in the coagulation cascade, clot formation, and wound healing process. Studies suggested that it also serves as biomarker of fibrinogen and inflammation and may be involved in the development of atherosclerosis [7–13]. A U-shaped association between platelet count and increased mortality has been recognized in the general population, women, the elderly, and patients with chronic obstructive pulmonary disease [14–17]. Studies also showed that platelet count was a prognostic indicator in the patients with stroke, and some infectious disease [18, 19]. In one study about the association about mean platelet volume (MPV) and mortality in incident HD patients, the author also found that lower baseline platelet counts were associated with higher mortality risk across all multivariable models [6]. Thrombocytopenia, defined as abnormally low platelet counts, may have serious consequences, such as increasing the risk of internal and external bleeding, delaying in wound healing and coagulation defects. Furthermore, HD patients might suffer from a prothrombotic adverse drug reaction called Heparin-induced thrombocytopenia (HIT) [20, 21]. However, the prevalence of thrombocytopenia in HD patients is understudied and the relationship between platelet counts and all-cause mortality and cardiovascular (CV) mortality in Chinese HD patients has not been previously explored.

Therefore, using data from China Dialysis Outcomes and Practices Pattern Study (DOPPS), we explored the prevalence of thrombocytopenia in chronic HD patients, and investigated the association between platelet counts and all-cause together with CV mortality.

## Methods

### Study design and subjects

The DOPPS is an international prospective cohort study of in-center adult HD patients which described in previous published papers [22, 23]. China joined DOPPS in 2011. DOPPS China randomly selected an average of 30 patients from 15 dialysis facilities in each city of Beijing, Shanghai, and Guangzhou. This was described in our previous study [24–26]. There were 1427 patients participated in China DOPPS5 (2012–2015). Of the 1427 patients, 58 patients were excluded from the present analysis as they didn’t have the platelet records. Baseline demographic and clinical data were collected at the start of participation in DOPPS5.

The authors confirm that all methods were carried out in accordance with relevant guidelines and regulations.

### Patient groups

Participants were divided into 2 groups according to their baseline platelet counts. Patients with thrombocytopenia (platelet< 100*10^9^/L) were assigned as TP group, and patients without thrombocytopenia as Non-TP group (platelet ≥100*10^9^/L). The Non-TP could not be further divided into normal (100*10^9^/L-300*10^9^/L) or above normal (platelet > 300*10^9^/L), since there were few patients who had platelet counts beyond the high-end of normal limits (300*10^9^/L).

### Outcomes

The primary end-point event was all-cause mortality. The secondary end-point event was the CV mortality during the follow-up period. We have a ‘Termination form’ to collect patients’ death information, including the date, place, primary reason, secondary reason of death. And the reasons of death were divided into several categories (cardiac, vascular, liver disease, infection, gastrointestinal, metabolic, other).

CV mortality was defined by the primary death records in the dataset. The following diagnosis in primary death records were considered as CV mortality: atherosclerotic heart disease, cardiac arrest, cardiac arrhythmia, cardiomyopathy, cerebro-vascular accident (including intracranial Hemorrhage), congestive heart failure, hemorrhage from ruptured vascular aneurysm, ischemic brain damage/anoxic encephalopathy, acute myocardial infarction, pulmonary embolus, stroke, and valvular heart disease.

### Statistics analysis

Continuous variables were represented as mean ± SD or median (25th, 75th) according to the results of normality test. Categorical variables were expressed as number and percentage. We stratified data by TP and Non-TP groups. Differences in mean or median among groups were evaluated by using analysis of variance or non-parametric test. Categorical data were compared using chi-square test.

Survival curves were produced by the Kaplan-Meier method and estimated by log-rank test. We used Cox proportional hazards models to assess the association of baseline platelet count with all-cause mortality, and CV mortality. All Cox models accounted for facility clustering effects by using the robust sandwich covariance estimate. Survival time for Cox models of all-cause mortality was the time from study entry to the end of study or to death, whichever occurred first. Similar calculation was taken for CV mortality. The Non-TP group was taken as the reference group for all analyses. Cox regression models were with 5 incremental levels of covariate adjustment. Model 1: unadjusted; model 2: adjusted for age, gender, body mass index (BMI), vintage; model 3: model 2 variables plus comorbidities (diabetes, coronary artery disease, congestive heart failure, other cardiovascular disease, cerebrovascular disease, hepatitis B and C, cancer (non-skin), peripheral vascular disease, lung disease, hypertension, psychiatric disorder, GI Bleeding, recurrent cellulitis, fracture, neurologic disease); model 4: model 3 plus hemoglobin, albumin, white blood cells, and serum creatinine; model 5: model 4 plus intradialytic weight loss, fistula use, primary kidney disease, standard kt/v, urine output< 200 ml/day.

We also used stepwise multivariate logistic analysis to identify the impact factors of TP. Odds ratio (OR) and 95% conference interval (CI) were calculated for each variable.

We performed MI procedure to impute missing data, and continuous and categorical variables were imputed 25 times by fully conditional specification regression and logistic regression, respectively. The imputed data sets were analyzed using the MI Analyze procedure in SAS/STAT 9.4. Percentages of missing for most variables were < 10%, except for single-pooled Kt/V (36.2%). *P* value < 0.05 was considered as statistically significant. All statistical analyses were performed with SAS, version 9.4 (SAS institute, Cary, NC; USA).

## Results

### Demographic data and clinical characteristics

There were 1369 patients had baseline platelet counts. In the study cohort, male patients were 54.8%. The median age was 60 (49, 71) years old and median dialysis vintage was 2.6 (0.9, 5.5) years. The median follow-up time of this study was 1.9 (1.2–2.1) years. The median platelet count was 160*10^9^ (123, 204). The baseline characteristics of HD patients was shown in Table 1. The prevalence of TP was 11.2%. Patients with TP tended to be with longer dialysis vintage, lower BMI, less likely had residual renal function (higher proportion of patients with urine output < 200 ml/day), lower Alb, lower white blood cells, and more likely having hepatitis and liver cirrhosis (Table 1).

**Table 1:** Baseline characteristics of HD patients according to the platelet counts

Variables	All	TP (*n* = 154)	Non-TP (*n* = 1215)	P value
**Demographics**				
Age (years)	60 (49, 71)	62 (50, 73)	60 (49, 71)	0.46
Males (%)	54.8	53.9	54.9	0.80
Vintage (years)	2.6 (0.9, 5.5)	3.87 (1.3, 7.9)	2.5 (0.9, 5.2)	< 0.01
BMI	21.9 ± 3.7	21.3 ± 3.4	20.0 ± 3.7	0.03
Urine output > 200 ml/day (%)	32.1	17.5	33.9	< 0.01
Primary kidney diseases (%)				0.17
Glomerulonephritis	39.2	43.5	38.7	
Diabetic nephropathy	23.3	16.9	24.1	
Hypertensive nephropathy	15.4	14.3	15.6	
others	22.1	25.3	21.7	
**Laboratory tests**				
Hgb (g/dl)	10.5 (9.3, 11.7)	10.5 (8.9, 11.7)	10.6 (9.3, 11.7)	0.98
Alb (g/dl)	3.9 (3.7, 4.2)	3.8 (3.6, 4.1)	4.0 (3.7, 4.2)	0.04
White blood cells (10^9/L)	6.0 (4.9, 7.3)	4.6 (3.8, 5.9)	6.1 (5.0, 7.4)	< 0.01
Creatine (mg/dl)	10.1 (8.0, 12.5)	9.6 (7.6, 11.9)	10.2 (8.1, 12.5)	0.07
**Dialysis prescription**				
spKt/V	1.4 (1.2, 1.6)	1.4 (1.2, 1.5)	1.4 (1.2, 1.6)	0.54
stdKtv_I	2.0 ± 0.3	2.1 ± 0.3	2.0 ± 0.4	0.27
Dialysis < 3 times /week (%)	21.0	20.1	21.1	0.83
Intradialytic weight loss (%)	0.04 (0.03, 0.05)	0.04 (0.03, 0.05)	0.04 (0.03, 0.05)	0.17
Fistula use (%)	85.0	85.7	84.9	1.00
**Comorbidities (%)**				
Diabetes	27.5	22.7	28.1	0.15
Coronary artery disease	25.3	25.3	25.3	0.92
Congestive heart failure	24.3	26.6	24.0	0.49
Other cardiovascular disease	21.3	22.7	21.1	0.68
Cerebrovascular disease	14.6	20.3	13.1	0.05
Hypertension	85.7	84.4	85.8	0.38
Peripheral vascular disease	9.4	9.7	9.3	0.88
Hepatitis	13.1	27.3	11.4	< 0.01
Lung disease	5.1	2.0	5.4	0.17
Cancer (non-skin)	3.9	4.6	3.9	0.66
GI Bleeding	3.9	2.3	2.5	0.26
Liver cirrhosis	1.3	5.5	0.8	< 0.01
All-cause death	14.7	26.0	13.3	< 0.01
Cardiac/Vascular death	7.5	11.7	6.9	0.05

Note: *BMI* body mass index, *Hgb* hemoglobin, *Alb* albumin, *spKt/V* single-pooled Kt/V, *stdKt/V* standardized Kt/V

### Associations between platelet counts and outcomes

**Fig. 1:**
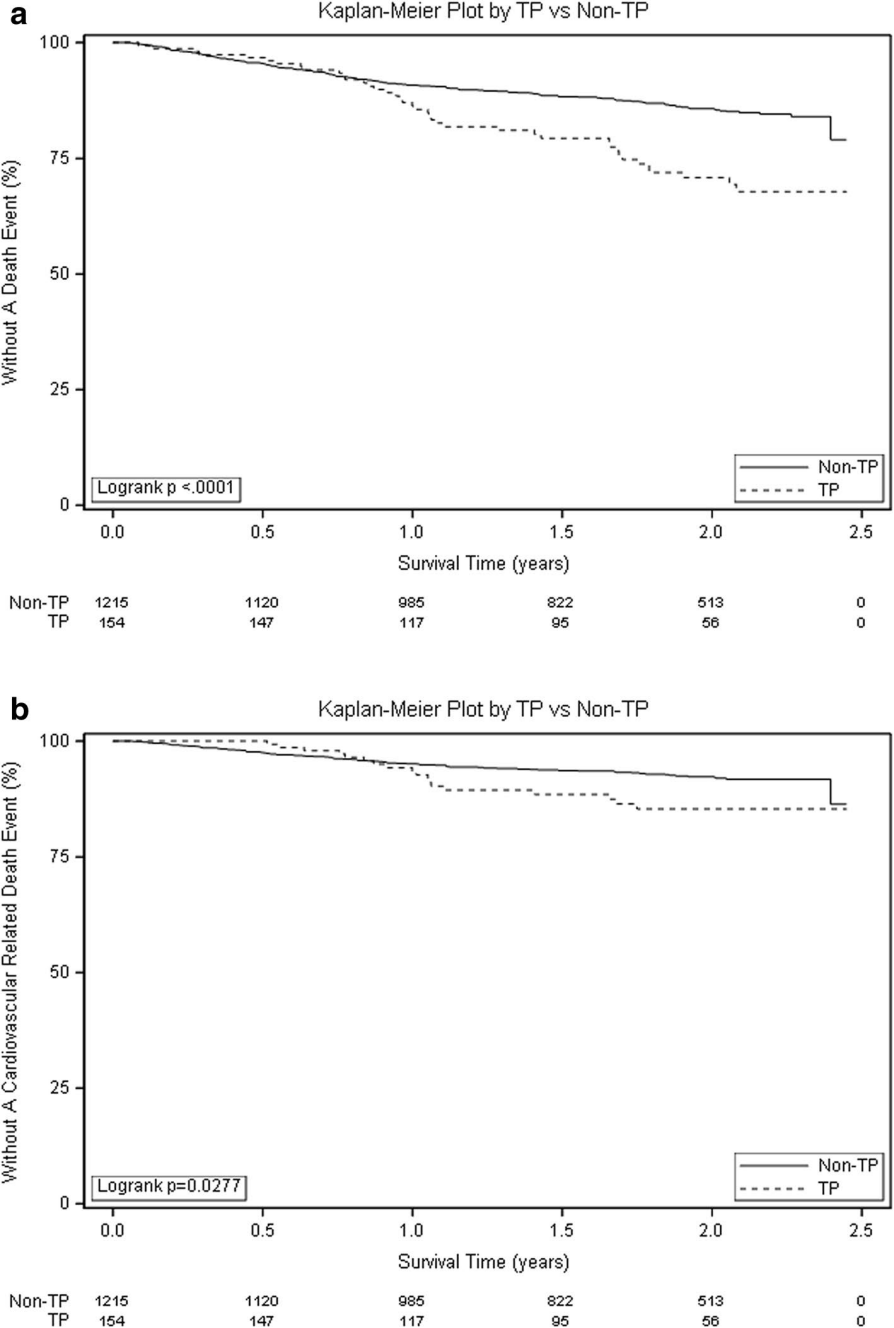
The Kaplan-Meier curves for TP and Non-TP groups in HD patients. **A** Survival curves of all-cause mortality; **B** Survival curves of CV mortality between two groups. Abbreviations: HD hemodialysis; TP thrombocytopenia; Non-TP without thrombocytopenia

**Fig. 2:**
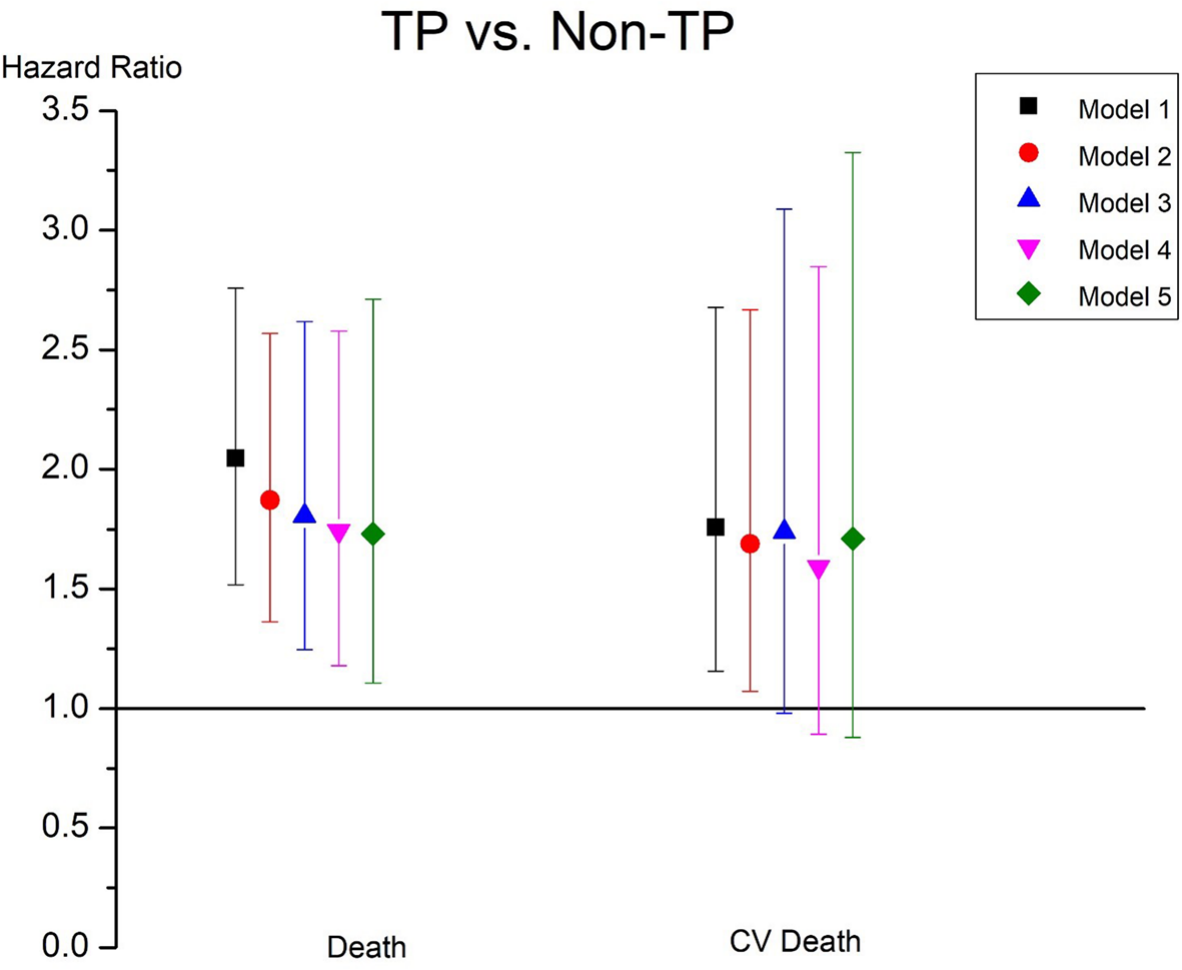
Associations between the platelet counts and all-cause mortality and CV mortality in different Cox regression models. Notes: Model 1: unadjusted; model 2: adjusted for age, gender, BMI, vintage; model 3: model 2 variables plus comorbidities (diabetes, coronary artery disease, congestive heart failure, other cardiovascular disease, cerebrovascular disease, hepatitis B and C, cancer (non-skin), peripheral vascular disease, lung disease, hypertension, psychiatric disorder, GI Bleeding, recurrent cellulitis, fracture, neurologic disease).; model 4: model 3 plus hemoglobin, albumin, white blood cells, and serum creatinine; model 5: model 4 plus Intradialytic weight loss, fistula use, primary kidney disease, standard kt/v, urine output < 200 ml/d. Abbreviations: TP thrombocytopenia; Non-TP without thrombocytopenia

Among 1369 included patients, 201(14.7%) died and 102 (7.5%) died from CV disease. The median platelet count in the alive group was 163*10^9^/L which was higher than that in the dead group which was 147*10^9^/L. The distribution of primary causes of death was shown in Table 2. According to the results of Kaplan-Meier analysis, patients with TP had significant higher risk of all-cause mortality and CV related deaths (log-rank test, *P* < 0.01 and 0.03 respectively, Fig. 1 A and B). In fully adjusted Cox model, TP were associated with higher all-cause mortality after adjusted for covariates (HR:1.73,95%CI:1.11,2.71). However, TP was not associated with CV deaths after full adjustment at the statistical significance level of 0.05 (HR:1.71,95%CI:0.88,3.33, Fig. 2).

**Table 2:** The distribution of primary causes of death

Causes of deaths (n, %)	All (*n* = 201)	TP (*n* = 40)	Non-TP (*n* = 161)
Cardiac/Vascular	102 (50.8)	18(45)	84(52.2)
Liver Disease	2 (1.0)	1(2.5)	1(0.6)
Infection	33 (16.4)	9(22.5)	24(14.9)
Gastrointestinal	8 (3.9)	1(2.5)	7(4.3)
Metabolic	6 (3.0)	0	6(3.7)
Other	19 (9.5)	3(7.5)	16(9.9)
Unknown	31 (15.4)	8(20)	23(14.3)

Note: Patients whose cause of death was missing was categorized into ‘unknown’ group. The OTHER cause including Bone marrow depression; Cachexia/failure to thrive; Malignant disease, patient ever on Immunosuppressive therapy; Malignant disease; Dementia, incl. Dialysis dementia, Alzheimer’s; Seizures; Chronic obstructive lung disease (COPD); Complications of surgery; Air embolism; Withdrawal from dialysis/uremia; Accident related to treatment; Accident unrelated to treatment; Suicide; Drug overdose (street drugs); Drug overdose; Multiple organ failure; Other cause of death

### Impact factors of thrombocytopenia

Stepwise multivariate logistic regression showed that urine output < 200 ml/day (OR: 2.01; 95% CI 1.29–3.15; *P* < 0.01), cerebral disease (OR: 1.63; 95% CI 1.00–2.66; P = 0.03), hepatitis (B or C, OR: 2.43; 95% CI 1.58–3.75; P < 0.01) were independent risk factor of TP. However, white blood cells were negatively associated with TP (OR: 0.70; 95% CI 0.62–0.78; P < 0.01, Table 3).

**Table 3:** Stepwise multivariate logistic regression for impact factors of thrombocytopenia

Variables	Odds ratios	95% CI	*P* value
Urine output < 200 ml/day (yes vs. no)	2.01	1.29–3.15	< 0.01
Cerebral disease (yes vs. no)	1.63	1.00–2.66	0.03
Hepatitis (B or C) (yes vs. no)	2.43	1.58–3.75	< 0.01
White blood cells	0.70	0.62–0.78	< 0.01

### Subgroup analysis

**Fig. 3:**
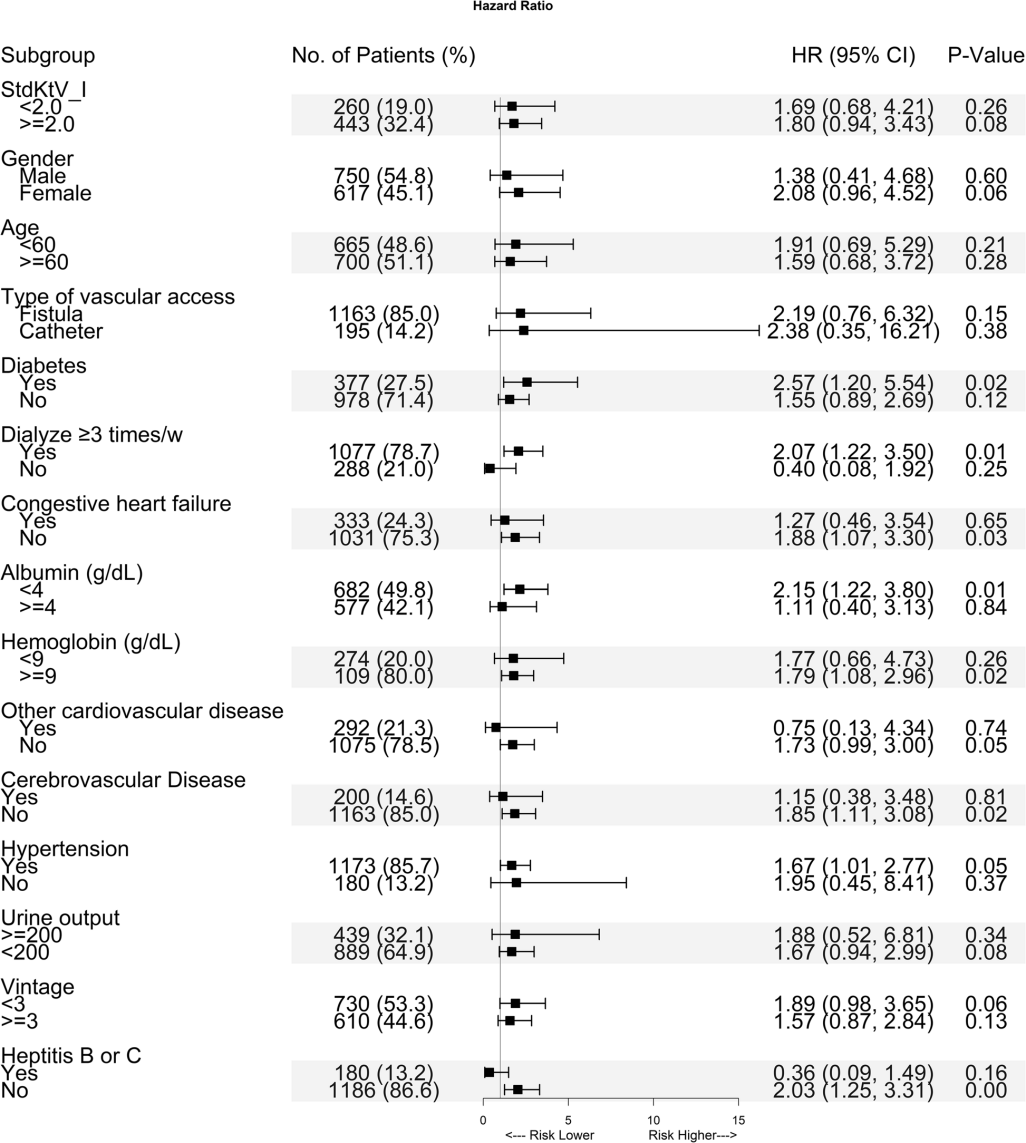
Association of thrombocytopenia with all-cause mortality across clinically relevant subgroups

Subgroup analysis found that the effect of TP on all-cause mortality was more prominent in patients with diabetes or hypertension, who on dialysis thrice a week, with lower ALB (< 4 g/dl) or higher hemoglobin, and those who without congestive heart failure, or cerebrovascular disease, hepatitis (*P* < 0.05, Fig. 3).

There seemed to have a trend but not of significant difference in patients with other cardiovascular disease (*p* = 0.05), and with dialysis vintage less than 3 years (*P* = 0.06).

There were no significant differences for patients who were younger or older (cutoff value: 60 years old), female or male, with or without adequate dialysis (standardized Kt/V, stdKtV > 2.0, with or without urine output < 200 ml, and using fistula or catheter.

## Discussion

In this large perspective cohort study of HD patients, we found that TP was positively associated with all-cause mortality. This finding is in line with similar observations made in incident dialysis patients [6] and patients with acute myocardial infarction/ acute coronary syndrome treated with percutaneous coronary intervention and acute ischemic stroke treated with mechanical thrombectomy [18]. The association between TP and CV mortality had a trend yet was not statistically significant after full adjustments. To our knowledge, this is the first study focused on investigating the association between platelet count and mortality in Chinese dialysis population. These findings expand upon our prior study suggesting TP might serve as a risk factor for all-cause mortality.

Like in the general and some other population, the previously mentioned retrospective study with large sample size found a U-shaped association in incident HD patients between platelet count and mortality [6]. However, the association between higher baseline platelet counts and death risk were attenuated by case-mixed adjustments [6] which was in line with another previously published research [5]. In that study, using national DaVita maintenance hemodialysis patient cohort, the author suggested that high platelet count might act as a link between renal cachexia and cardiovascular mortality [5]. In our study, we could not explore if high-platelet counts (thrombocytosis) will be associated with mortality in our cohort as limited by the small number of patients with platelet count higher than the upper limit.

The causative link between TP and increased mortality risk is not entirely clear. TP related to hemorrhagic complications which could be one plausible explanation. Administration of heparin in the dialysis process might lead to an autoantibody-mediated destruction of platelets, called HIT [20, 21]. Some HIT patients may show unusual clinical consequences, such as thrombocytopenia, disseminated intravascular coagulation and microvascular thrombosis. Several studies showed that HD patients with HIT were at a higher risk of cardiovascular mortality and arteriovenous fistula thrombosis than patients without HIT [20, 21, 27]. Some studies showed that in immune thrombocytopenic purpura and stoke patients, elevated levels of platelet microparticles (PMP) might be associated with an increased thrombogenic risk hence mortality risk [28–30]. Whether PMP has any influence on adverse outcomes in HD patients is worthy of further study.

Studies in healthy populations reported contradict results about the association between platelet concentrations and function with cardiovascular mortality [31, 32]. In a cohort of incident peritoneal dialysis patients, researchers found higher platelet counts and plateletcrit might be associated with higher risk for cardiovascular mortality [33]. In our study, we found the trend that TP might be associated with cardiovascular mortality but was not of statistically significance. However, the number of cardiovascular events was small in our cohort.

Trying to decipher the underlying pathophysiology of TP is challenging. Not surprisingly, some comorbidities, such as cerebral disease and hepatitis are positively associated with higher risk for TP. Liver cirrhosis is one of the major causes of low platelet count in clinical settings. We did have a higher proportion of liver cirrhosis in TP group than in Non-TP group patients. However, there were all together 18 patients (1.3%) with liver cirrhosis in this cohort which should not be the main underlying cause. Auto-immune diseases and bone marrow diseases might also affect platelet counts. In primary causes of ESKD, these should be categorized as other causes which had similar proportion in both TP and Non-TP patients. However, we could not identify these diseases in patients’ comorbidities. We found that white blood cells were negatively associated with TP. Both of platelet and white blood cell might be influenced by the bone marrow status. Why urine output < 200 ml/day was associated with higher risk of TP in our patients is not fully understand. As noted, drugs may influence blood platelet counts, however, we could not do further analysis due to the limited data about drug usage.

Our study has several limitations. Firstly, this is an observational study which might has inherent shortcomings such as selection bias and confounding factors. Secondly, the study was lack of additional measurements of platelet activity and function, such as MPV and PMP. Thirdly, we could not identify the proportion of patients who had auto-immune diseases and bone marrow diseases which might have influence on platelet counts and mortality. Finally, we could not do analysis related with drug usage. Nevertheless, we believe these factors should not affect our main findings.

## Conclusion

In this prospective cohort study, thrombocytopenia was associated with an increased risk of all-cause mortality. The association was not attenuated by adjusting for several potential confounding factors. Platelet counts may be used as early available outcome predictors among HD patients.

## Data Availability

The data used of this study are available from the corresponding author on reasonable request.

## Abbreviations

HD: Hemodialysis
TP: Thrombocytopenia
CV: Cardiovascular
DOPPS: Dialysis Outcomes and Practice Patterns Study
ESKD: End stage kidney disease
MPV: Mean platelet volume
HIT: Heparin-induced thrombocytopenia
BMI: Body mass index
stdKt/V: Standardized Kt/V
OR: Odds ratio
CI: Conference interval
PMP: Platelet microparticles
